# Outcomes and CT Perfusion Thresholds of Mechanical Thrombectomy for Patients With Large Ischemic Core Lesions

**DOI:** 10.3389/fneur.2022.856403

**Published:** 2022-06-01

**Authors:** Hongchao Yang, Dinglai Lin, Xiaohui Lin, Yanmin Wu, Tingyu Yi, Wenhuo Chen

**Affiliations:** ^1^Department of Neurosurgery, Beijing Chaoyang Hospital, Capital Medical University, Beijing, China; ^2^Department of Neurology, Zhangzhou Affiliated Hospital of Fujian Medical University, Fujian, China

**Keywords:** acute ischemic stroke, mechanical thrombectomy, CT perfusion, large ischemic core, cerebral blood flow

## Abstract

**Objective:**

To explore the clinical prognosis and factors after mechanical thrombectomy (MT) in patients with large cerebral infarction assessed by computed tomographic perfusion (CTP)and the optimal threshold of cerebral blood flow (CBF) for estimating ischemic core.

**Methods:**

We analyzed data from the anterior circulation database of our hospital (August 2018–June 2021). Multivariate logistic regression analyses identified the predictors of clinical outcomes for patients with large baseline infarcts (>50 ml) assessed by the MIStar software. The receiver operating characteristic (ROC) analysis was used to explore the cutoff value of factors.

**Results:**

The present study included one hundred thirty-seven patients with large baseline infarcts. Moreover, 23 (16.8%) patients achieved functionally independent outcomes, and 50 (36.5%) patients died at 90 days. A total of 20 (14.7%) patients had symptomatic intracranial hemorrhage (sICH). The multivariable analysis showed that higher age and larger core volume were independent of poor outcomes. The cutoff value of core volume was 90 ml, and the age was 76 years. Hypertension and rt-PA treatment were independent factors of sICH. Higher age and larger ischemic volume were independent risk factors of mortality.

**Conclusions:**

Mechanical thrombectomy can be applied in patients with large ischemic core volumes. Patients older than 76 years with large cores (>90 ml) are unlikely to benefit from MT. These findings may be helpful in selecting patients with large baseline infarcts to be treated by MT. The threshold of CBF < 30% is the independent factor, and this is worth evaluating in future studies to find the optimal threshold of CBF.

## Introduction

Mechanical thrombectomy (MT) has demonstrated its safety and efficacy in selected patients with acute ischemic stroke (AIS) due to anterior proximal vessel occlusion (PVO) (1–4). However, patients with large ischemic core (≥50 or ≥70 ml) (1) and computed tomography (CT)-based-Alberta Stroke Program Early CT score (ASPECTS < 6) were excluded in previous randomized clinical trials that demonstrated the validation of MT (5).

Some studies reported that patients with large ischemic core strokes had lower functional independence rates (5, 6). However, some studies have also shown that MT can improve the clinical prognosis of patients with a large ischemic core (7). So, it is controversial for patients with large core infarction treated with MT.

Computed tomographic perfusion (CTP) has been used to assess ischemic penumbra and core infarction (8). In addition, previous studies have suggested that CTP can be used as a tool to select patients who may benefit from MT. (9, 10). The cerebral blood flow (CBF) < 30% has been used to estimate the ischemic core in previous studies. However, the CBF < 30% may overestimate the ischemic core volumes, potentially leading to some patients losing the opportunity of MT. (11). So, the optimal threshold for estimating the ischemic core is still unclear.

Therefore, we explored the clinical prognosis and factors after MT in patients with large acute area cerebral infarction assessed by CTP and the optimal threshold of CBF for estimating ischemic core.

## Methods

### Study Design and Ethics

The study was an observational retrospective cohort study. We recorded patients' clinical and imaging data, such as demographics, past medical history, symptomatic intracranial hemorrhage, and 3-month clinical prognosis.

The data were obtained from the anterior circulation database of a comprehensive stroke center. The patient or the proxy signed the informed consent.

### Inclusion Criteria

The inclusion criteria for the present study were as follows: acute occlusion of anterior circulation vessel; time stroke onset to treatment <24 h; age ≥18 years; the baseline National Institutes of Health Stroke Scale (*n*IHSS) score ≥ 5; pretreatment ischemic core volume ≥50 ml (assessed by CBF < 30%); and premorbid modified Rankin Scale (mRS) score ≦3. There are no exclusion criteria.

### Imaging Analysis and Mismatch Definition

Patients were scanned using a 256-slice CT scanner (Revolution; GE Healthcare, Waukesha, WI). For the CTP (70 kV peak, 225 mA, 2.0 s cycle time, 22 cm recon field of view (FoV), and 12 cm coverage) protocol, 45 ml CT contrast agent was power injected at 4.5 ml/s followed by a saline chase of 40 ml at 6 ml/s.

Perfusion maps and ischemic core volumes (CTP volumes) were determined using MIStar software (Apollo Medical Imaging Inc, Melbourne, VIC, Australia). Delayed time (DT > 3 s) was defined as the hypoperfusion brain tissue, and the ischemic core volumes were relative CBF (rCBF) < 30%, rCBF < 25%, and rCBF < 20%. The mismatch volume (volume of DT > 3 s minus volume of rCBF) and mismatch ratio (volume of DT > 3 s divided by volume of rCBF < 30%, rCBF < 25%, and rCBF < 20%) were also calculated by MIStar software.

### Assessment Criteria

The assessment of clinical prognosis was the use of the mRS score at 3 months, and the definition of functional independence outcome was a score of 2 or less. The definition of a favorable outcome was the mRS score of 3 or less. The assessment of reperfusion was the modified treatment in cerebral infarction (mTICI) scale, and the substantial reperfusion was the state of 2b, 2c, or 3. The secondary outcomes, such as 3-month mortality and sICH within 7 days, were also assessed. The definition of sICH was according to the Heidelberg Bleeding Classification (12).

### Statistical Analysis

Continuous data were presented as mean ± SD and categorical data as frequency and percentage. Analysis was carried out with an independent-samples *t*-test and the chi-square and Fisher's exact tests.

Univariate logistic regression was used to analyze potential factors, and a covariate with a univariate *p-value* < 0.05 was included in multivariate logistic regression to identify predictors of clinical outcomes. The odds ratio (*OR*), 95% confidence interval (*CI*), and *p-value* were determined for factors of the univariate and multivariate models. The receiver operating characteristic (ROC) analysis was used to explore the cutoff value of factors.

Statistical significance was defined as *p* < 0.05 and an *OR* with a 95% *CI*. Statistical analysis was carried out with SPSS version 22.0 (SPSS Inc, Chicago, Illinois).

## Results

### Populations and Baseline Characteristics

Between August 2018 and June 2021, we reviewed 137 patients with large ischemic cores due to large vessel occlusion of anterior circulation treated by mechanical thrombectomy (46.7% women and mean age 69.5 ± 10.4 years) (as shown in Table 1 for the baseline clinical characteristics) (Supplementary Figure 1 shows the patients' flow diagram.). Patients without functional independence outcomes were more elderly than those in the functional independence outcomes group (65.5 ± 10.8 vs. 70.3 ± 10.1, *p* = 0.042). The functional independence outcome group had fewer women (21.7 vs. 51.8%, *p* = 0.008). Patients without functional independence outcomes were more likely to have middle cerebral artery (MCA) and internal carotid artery (ICA) terminus occlusion (*p* = 0.007). There was no significance difference in time from onset to treatment between the functional group and without functional group.

**Table 1:** Baseline clinical characteristics of different groups.

**Variable**	**Total (*n =* 137)**	**With functional independence (mRS0-2, *n =* 23)**	**Without functional independence (mRS3-6, *n =* 114)**	***P***
Age (year) (mean ±SD)	71.2 ± 12.1	60.0 ± 13.1	73.5 ± 10.6	<0.001
Female sex (*n* %)	64(46.7)	5(21.7)	59(51.8)	0.008
Hypertension (*n* %)	96(70.1)	14(60.9)	82(71.9)	0.291
Diabetes mellitus (*n* %)	45(32.8)	6(26.1)	39(34.2)	0.449
Hyperlipidemia (*n* %)	20(14.6)	5(21.7)	15(13.2)	0.331
Atrial fibrillation (*n* %)	30(21.9)	2(8.7)	28(24.6)	0.093
History of stroke (*n* %)	2(1.5)	1(4.3)	1(0.9)	0.309
Tobacco use (current or past) (*n* %)	30(21.9)	5(21.7)	25(21.9)	0.984
Occlusion artery				0.007
M1 (*n* %)	57(41.6)	6(26.1)	51(44.7)	
ICA terminus (*n* %)	64(46.7)	10(43.5)	54(47.4)	
Tandem (*n* %)	16(11.7)	7(30.4)	9(7.9)	
NIHSS (mean ±SD)	16.8 ± 5.9	16.3 ± 5.3	16.9 ± 6.0	0.661
Stroke etiology(*n* %)				0.458
Atrial fibrillation	31(22.6)	4(17.4)	27(23.7)	
Atherosclerosis	71(51.8)	12(52.2)	59(51.8)	
Dissection	14(10.2)	2(8.7)	12(10.5)	
Other	10(7.3)	3(13.0)	7(6.1)	
Undetermined	11(8.0)	2(8.7)	9(7.9)	
Baseline ASPECTS (mean ±SD)	3.1 ± 2.6	3.5 ± 2.8	2.8 ± 2.0	0.281
iv tPA (*n* %)	50(36.5)	11(47.8)	39(34.2)	0.216
mTICI score of 2b-3 (*n* %)	122(89.1)	21(91.3)	101(88.6)	1.0
**Time from onset to perfusion scan, min (mean** **±SD)**				
Time from onset to treatment, min (mean ±SD)	424.8 ± 762.9	344.5 ± 314.3	441.7 ± 826.7	0.581
Time from groin puncture to reperfusion, min. (mean ±SD)	61 ± 40	60 ± 40	67 ± 42	0.478
sICH (*n* %)	20(14.6)	3(13.0)	17(14.9)	1.0
**CBF** **<** **30%**				
Ischemic core, ml (mean ±SD)	96.9 ± 47.1	68.2 ± 11.0	102.7 ± 49.4	0.001
Mismatch volume ml (mean ± SD)	123.8 ± 105.2	125.8 ± 113.1	113.8 ± 50.7	0.62
Mismatch ratio (mean ±SD)	2.5 ± 1.5	2.7 ± 0.7	2.4 ± 1.6	0.42
**CBF < 25%**				
Ischemic core, ml (mean ±SD)	79.8 ± 43.7	53.8 ± 11.5	85.1 ± 45.9	0.002
Mismatch volume, ml (mean ±SD)	140.8 ± 106.9	143.5 ± 114.7	127.8 + 50.2	0.52
Mismatch ratio (mean ±SD)	3.1 ± 2.1	3.1 ± 2.2	2.5 ± 1.2	0.36
**CBF** **<** **20%**				
Ischemic core, ml (mean ±SD)	62.5 ± 39.1	39.5 ± 12.0	67.2 ± 41.0	0.003
Mismatch volume, ml (mean ±SD)	158.2 ± 108.4	161.5 ± 116.1	141.7 ± 55.1	0.43
Mismatch ratio (mean ±SD)	4.3 ± 3.3	5.1 ± 2.3	4.1 ± 3.4	0.22

### Ischemic Core and Penumbra

The ischemic core volume based on CTP was 96.9 ± 47.1 ml (CBF < 30%), 79.8 ± 43.7 ml (CBF < 25%), and 62.5 ± 39.1 ml (CBF < 20%).

Compared with the functional independence outcome group, the ischemic core volume was lager in the non-functional independence outcome group (CBF < 30%: 102.7 ± 49.4 vs. 68.2 ± 11.0 ml, *p* = 0.001; CBF < 25%: 85.1 ± 45.9 vs. 53.8 ± 11.5 ms, *p* = 0.002; and CBF < 20%: 67.2 ± 41.0 vs. 39.5 ± 12.0 ml, *p* = 0.003). The mismatch volume and ratio were not different in the two groups. The large core volume (≥70 ml) was in 68.6% (94/137) patients (mean core volumes: 114.4 ± 47.5 ml, mean age: 72.7 ± 12.3 years).

### Outcomes

At a 3-month follow-up, 23(16.8%) patients were functionally independent. The factors of patients without functional independence were higher age, being female, occlusion artery of MCA and ICA terminus, and higher ischemic core volumes. The multivariable analysis showed that higher age and larger ischemic core volume (calculated by CBF < 30%) were independent factors of poor functional outcomes.

For patients with mRS 0–3 as the dependent variable, 25.5% (35/137) of patients had a favorable outcome at a 3-month follow-up. The unfavorable outcome was associated with higher age, occlusion artery of MCA and ICA terminus, larger ischemic core volume (calculated by CBF < 30%), atrial fibrillation, and higher NIHSS (Supplementary Table 1).

The substantial recanalization was achieved in 122 (89.1%) patients and was not different in groups (functional independence group vs. non-functional independence group: 91.3 vs. 88.6%, *p* = 1.0).

A total of 20 (14.7%) patients had sICH, including 3 (13.0%) patients with functional independent and 17 (14.9%) patients with poor outcome (*p* = 1.0). In the present study, factors associated with sICH were hypertension, hyperlipidemia, and the use of tobacco and recombinant tissue plasminogen activator (rt-PA) (Supplementary Table 2). Hypertension and the use of rt-PA treatment were independent factors of sICH (Table 2).

**Table 2:** Multivariate logistic regression analysis for outcomes.

**Variable**	**OR**	**95%CI**	***P***
**A. With mRS 0–2 as the dependent variable**			
Age	1.11	1.04–1. 18	0.001
CBF < 30%, ml	1.06	1.02–1. 11	0.010
**B. With mRS 0–3 as the dependent variable**			
Age	1.08	1.03–1. 12	0.001
CBF < 30%, ml	1.02	1.00–1. 04	0.033
Atrial fibrillation	4.3	1.09-17.3	0.038
**C.With sICH as the dependent variable**			
Hypertension	20.09	5.01–80.55	<0.001
iv tPA	8.9	2.31–34.86	0.023
**D. With mRS 6 as the dependent variable**			
Age	1.07	1.03–1. 12	0.001
CBF < 30%, ml	1.01	1.0–1. 02	0.047

At a 3-month follow-up, the mortality rate was 36.5% (50/137). The multivariable analysis considered higher age and larger ischemic core volume (calculated by CBF < 30%) as independent risk factors of mortality (Table 2).

**Figure 1:**
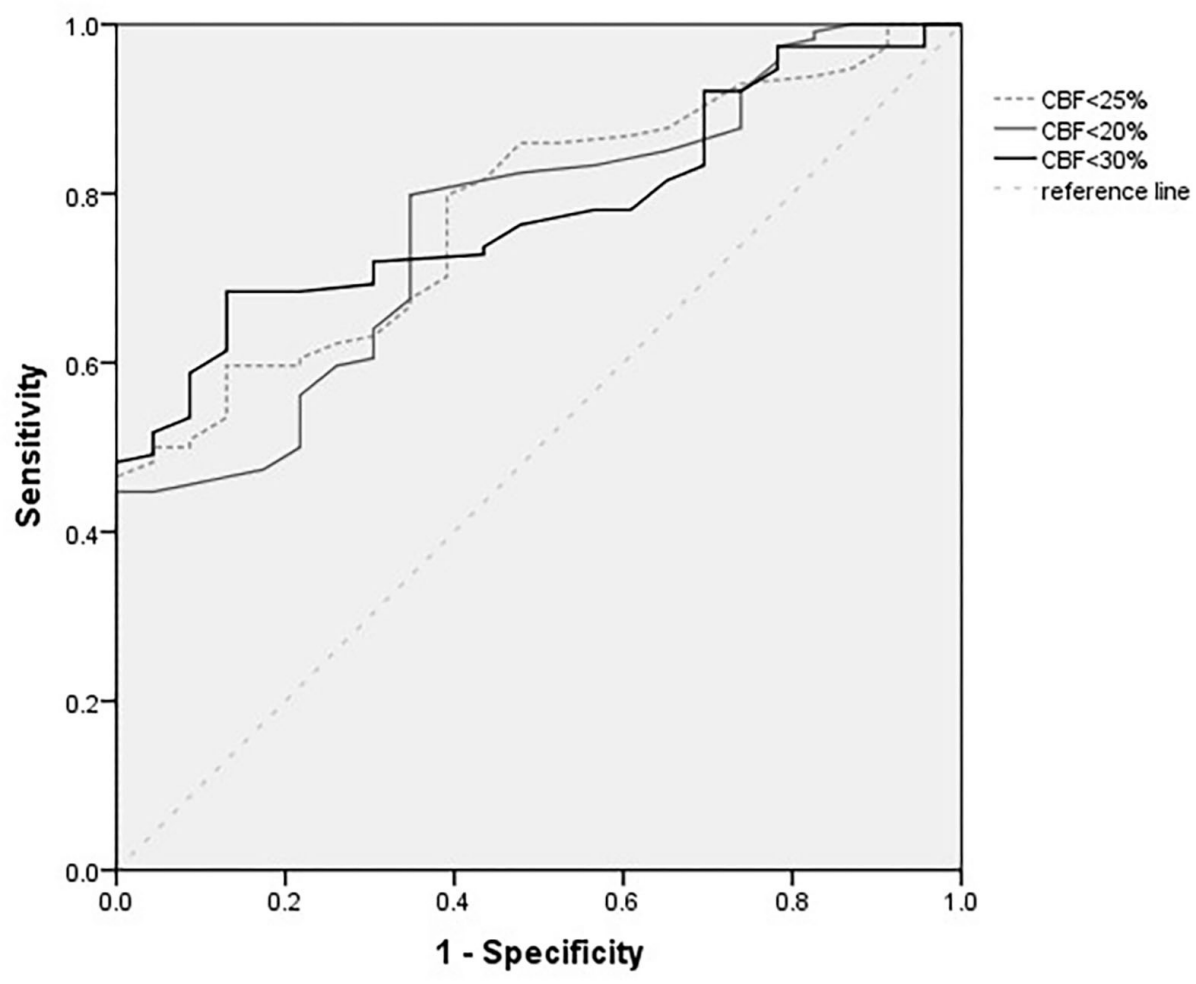
Receiver operating characteristic (ROC) curves for different definitions of core infarcts volumes (ml).

Figure 1 shows the ROC analysis for the prediction of functional independence outcome by the ischemic core volumes (calculated by CBF < 30%, CBF < 25%, and CBF < 20%). The volume threshold of CBF < 30% for the highest sensitivity (0.68) and specificity (0.87) was 75 ml. The cutoff value of ischemic core volume (CBF < 25% and CBF < 20%) were 65 and 40 ml, respectively. The highest sensitivity of CBF < 25% and CBF < 20% was 0.60 and 0.65, and the specificity was 0.87 and 0.76.

**Figure 2:**
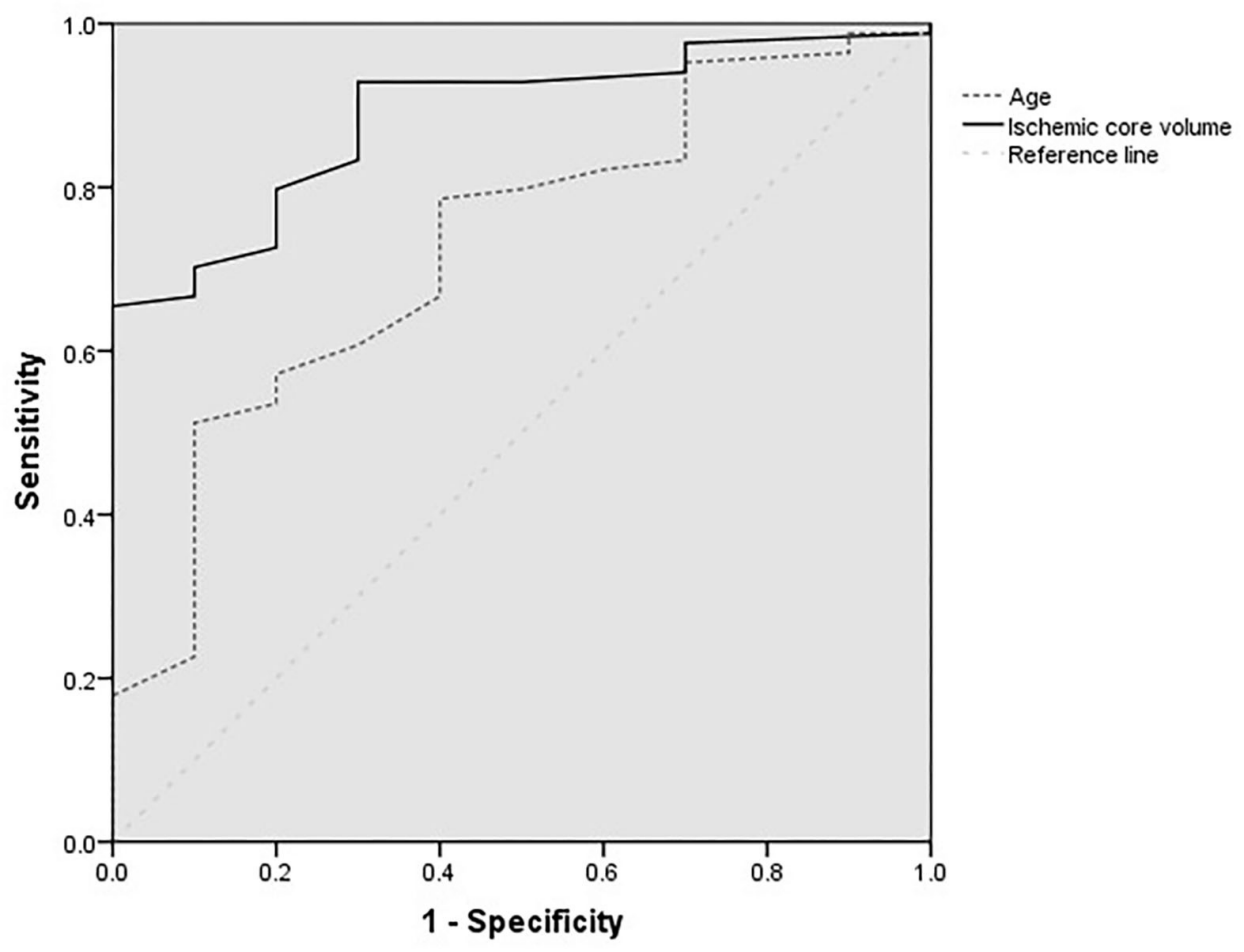
Receiver operating characteristic curves for age (year) and ischemic core volumes of patients with baseline core volumes larger than 70 ml.

Figure 2 shows the ROC analysis for the prediction of functional independence outcome by the ischemic core volumes (calculated by CBF < 30%, ≥ 70 ml) and age. The cutoff value of core volume was 90 ml, and the age was 76 years old. The highest sensitivity of these two factors was 0.66 and 0.51, and the specificity was 0.97 and 0.90, respectively.

## Discussion

The prognosis of patients with large baseline ischemic core volume treated by MT is still uncertain. In the present study, we found that 16.8% (23/137) of patients achieved functional independence outcome, and 36.5% of patients died at a 3-month follow-up, which showed a worse outcome at 3 months after stroke than patients with small infarcts in the previous studies (5, 6, 13). Our findings again confirmed that patients with large baseline ischemic core volume were associated with poor outcomes at 3 months after stroke. However, there were still 25.5% (35/137) of patients with a favorable outcome at a 3-month follow-up in the present study. So, we tried to find the factors of prognosis in our study, which may help guide MT for large baseline infarct in clinical practice.

In the present study, we used the CTP to assess the large baseline ischemic core volume and was consistent with previous studies (9, 10).

Bandera reported that the optimal CBF varied widely in their review study (14). Campbell also reported that the optimal threshold was <31% of the mean contralateral CBF (15). However, the ischemic core volume (defined by CBF < 30%) may be overestimated (11). The CBF < 30% may not be the optimal threshold for assessing the ischemic core volume, especially in a very early time window (16, 17). Saraji reported that they planned to use the CBF < 20% threshold in patients within a 2 h time window after stroke in the study of SELECT2 (18). Therefore, we also estimated the ischemic core volume by CBF < 30%, CBF < 25%, and CBF < 20%, which tried to find an appropriate definition of ischemic core volume. In the present study, we used these three definitions to assess the ischemic core volume, and all of them were related to prognosis in univariate analysis. However, only the ischemic core volume estimated by CBF < 30 was the independent risk factor. The result may be affected by the time from onset to treatment, which was more than 120 min in our study. In future research, a more appropriate algorithm may be required to determine the definition of infarction core.

In the present study, we found that larger baseline ischemic core volumes were the independent factor of the prognosis, which was in line with the results of Panni's study (19). Yoshimoto reported that patients with 70 and 100 ml ischemic volumes might benefit from MT, and the upper core limit is approximately 120 ml (20). In the present study, we further analyzed the patients with core infarction volume larger than 70 ml by ROC analysis. We found that patients with a core infarction volume larger than 90 ml had worse outcomes at a 3-month follow-up, which implies that patients with large cores (>90 ml) may not benefit from MT.

Our study also found that higher age was the independent factor of poor outcomes. Mourand and Danière reported that the cutoff value of 70 years was the treatment consideration, and patients younger than 70 years had a better outcome after MT (21, 22). In our study, the ROC analysis showed that patients over 76 years old had a worse outcome, which means that patients older than 76 years with large cores may not benefit from MT.

Gilgen reported that the rate of sICH in patients with large cores (>70 ml) was 16.1% (13), which was consistent with our study (sICH: 14.6%). No significant difference emerged in our study between patients with functional independence outcomes and patients without independence outcomes. However, the Highly Effective Reperfusion evaluated in Multiple Endovascular Stroke Trials (HERMES) study suggests a higher rate of sICH in patients with large volume infarcts treated by MT. So, it is worth looking for factors of sICH. In our study, hypertension was the independent factor of sICH. Chen also reported that patients with higher hypertension were more likely to have sICH (23). More studies are needed to evaluate the factors and find the cutoff value of hypertension. The randomized trials of intravenous Alteplase before MT in Asia and Europe showed that the percentage of sICH was similar between the two groups, which means that the use of rt-PA before MT did not increase the risk of sICH more than that of direct MT (24, 25). However, we found that intravenous Alteplase before MT increased the risk of sICH in the present study, which may be associated with large baseline infarcts. The factors of sICH and bridging thrombectomy for large baseline infarcts after thrombolysis are required to be studied.

In the present study, the 3-month mortality rate was 36.5%, which was in line with the results (mortality rate: 31.5%) of Kerleroux's study (26). The factors of morality were larger volume infarcts and higher age. Many studies have reported that patients with larger infarct volume and higher age were more likely to die at a 3-month follow-up (17, 27). Our study again confirmed that these factors were associated with poor outcomes. Kaesmacher reported that patients with successful reperfusion were independently associated with reduced mortality (28). Despite the fact that we did not find that successful recanalization is a statistical factor for a good prognosis. However, patients with favorable outcomes had a higher percentage of successful recanalization in our study. This means that a tendency for successful recanalization may have lower mortality rates in patients with large baseline infarcts. More studies are required to confirm the results.

## Limitations

The present study has several limitations. First, this is a retrospective study that leads to a selection bias. Second, the follow-up time is only 3 months, and the functional independence outcome may be underestimated. Third, we used the software of MIStar to assess the core volume, which may differ between different software packages, such as RAPID software, though previous studies have confirmed the efficacy of MIStar in the use of assessing core volume (29).

## Conclusion

The present study showed that MT could be applied in patients with large ischemic core volumes. Patients older than 76 years with large cores (>90 ml) are unlikely to benefit from MT. The threshold of CBF < 30%, CBF < 25%, and CBF < 20% are used to estimate core volume in this study, and only the threshold of CBF < 30% is the independent factor. This is worth evaluating in future studies to find the optimal threshold of CBF.

## Data Availability Statement

The raw data supporting the conclusions of this article will be made available by the authors, without undue reservation.

## Ethics Statement

The studies involving human participants were reviewed and approved by the Ethics Committees of Zhangzhou Shi Hospital. The patients/participants provided their written informed consent to participate in this study.

## Author Contributions

HY: substantial contributions to the conception and design of the work, acquisition, analysis, and interpretation of data, and drafting the work and revising it critically for important intellectual content. DL and YW: analysis and interpretation of data. XL: acquisition of data. TY and WC: substantial contributions to the conception and design of the work, analysis and interpretation of data, and drafting the work and revising it critically for important intellectual content. All authors gave their final approval of the version published and have agreed to be accountable for all aspects of the work in ensuring that questions related to the accuracy or integrity of any part of the work are appropriately investigated and resolved.

## Funding

This study was supported by the National Health Commission Capacity Building and Continuing Education Center GWJJ2021100203.

## Conflict of Interest

The authors declare that the research was conducted in the absence of any commercial or financial relationships that could be construed as a potential conflict of interest.

## Publisher's Note

All claims expressed in this article are solely those of the authors and do not necessarily represent those of their affiliated organizations, or those of the publisher, the editors and the reviewers. Any product that may be evaluated in this article, or claim that may be made by its manufacturer, is not guaranteed or endorsed by the publisher.
