# Supplementary figures and images for: Outcomes and CT Perfusion Thresholds of Mechanical Thrombectomy for Patients With Large Ischemic Core Lesions
Source: Front Neurol. 2022 Jun 1;13:856403. doi: 10.3389/fneur.2022.856403 (PMC9198314; doi:10.3389/fneur.2022.856403)

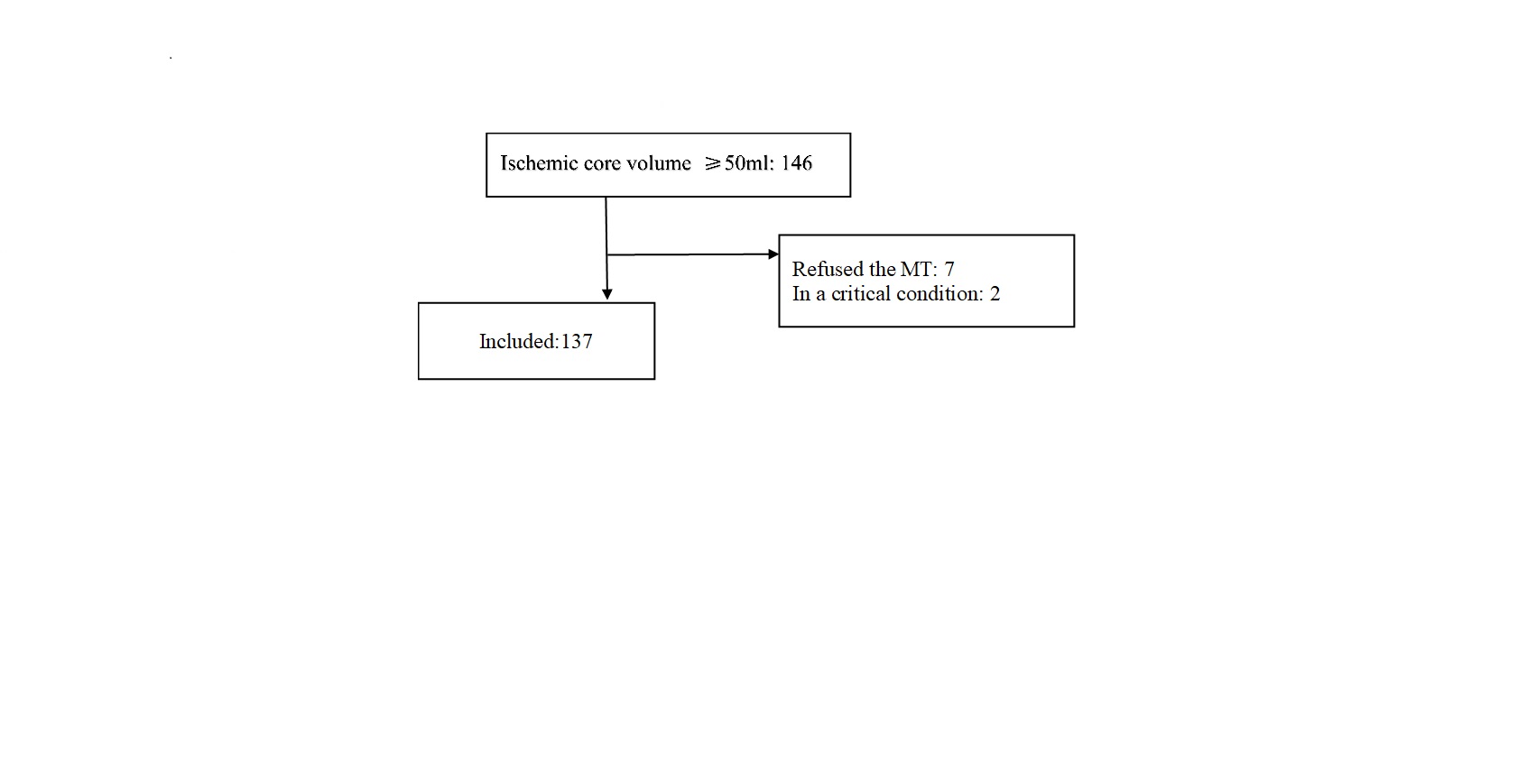

Supplement: Supplementary file 1 [file Image_1.JPEG]
