# Supplementary material for: Outcomes and CT Perfusion Thresholds of Mechanical Thrombectomy for Patients With Large Ischemic Core Lesions
Source: Front Neurol. 2022 Jun 1;13:856403. doi: 10.3389/fneur.2022.856403 (PMC9198314; doi:10.3389/fneur.2022.856403)
Supplement: Supplementary file 2 [file Table_1.docx]

**Supplement Tables**

| Table 1 Baseline clinical characteristics of different groups | | | |
| --- | --- | --- | --- |
| variable | outcome of patients with mRS 0-3( n=35) | outcome of patients with mRS4-6( n=102) | P |
| Age(year) (mean ±SD) | 62.1±13.3 | 74.4±10.0 | <0.001 |
| Female sex (n %) | 9(25.7) | 55(53.9) | 0.004 |
| Hypertension (n %) | 22(62.9) | 74(72.5) | 0.28 |
| Diabetes mellitus (n %) | 9(25.7) | 36(35.3) | 0.298 |
| Hyperlipidemia (n %) | 9(25.7) | 11(10.8) | 0.331 |
| Atrial fibrillation (n %) | 3(8.6) | 27(26.5) | 0.027 |
| History of stroke( n %) | 1(2.9) | 1(1.0) | 0.447 |
| Tobacco use (current or past) (n %) | 7(20.0) | 23(22.5) | 0.753 |
| Occlusion artery |  |  | <0.001 |
| M1 (n %) | 19(54.3) | 38(37.3) |  |
| ICA terminus (n %) | 9(25.7) | 55(53.9) |  |
| Tandem (n %) | 7(20.0) | 9(8.8) |  |
| NIHSS (mean ±SD) | 17.5±5.6 | 19.7±4.6 | 0.022 |
| Baseline ASPECTS (mean ±SD) | 3.4±2.6 | 3.2±2.7 | 0.83 |
| iv tPA (n %) | 15(42.9) | 35(34.3) | 0.365 |
| mTICI score of 2b-3 (n %) | 31(88.6) | 91(89.2) | 1.0 |
| Time from onset to treatment, min (mean ±SD) | 363.4±395.7 | 439.6±817.3 | 0.2 |
| Time from groin puncture to reperfusion, min. (mean ±SD) | 53±31 | 63±43 | 0.198 |
| sICH (n %) | 3(8.8) | 17(16.7) | 0.263 |
| CBF<30% |  |  |  |
| Ischemic core, ml(mean ±SD) | 76.5±28.1 | 105.2±49.3 | 0.001 |
| Mismatch volume ml(mean ±SD) | 132.7±117.7 | 107.4±47.2 | 0.22 |
| Mismatch ratio(mean ±SD) | 2.6±1.8 | 2.5±0.7 | 0.733 |
| CBF<25% |  |  |  |
| Ischemic core, ml(mean ±SD) | 62.3±26.0 | 87.2±46.0 | 0.003 |
| Mismatch volume, ml(mean ±SD) | 146.3+119.8 | 125.0±51.9 | 0.311 |
| Mismatch ratio(mean ±SD) | 3.3±2.8 | 3.1±1.1 | 0.647 |
| CBF<20% |  |  |  |
| Ischemic core, ml(mean ±SD) | 47.8±24.2 | 68.9±40.9 | 0.005 |
| Mismatch volume, ml(mean ±SD) | 166.6±121.8 | 136.8±50.5 | 0.163 |
| Mismatch ratio(mean ±SD) | 4.7±5.0 | 4.3±2.1 | 0.611 |

| Table2 Factors of sICH | | | |
| --- | --- | --- | --- |
| variable | With sICH ( n=20) | Without sICH( n=117) | P |
| Age(year) (mean ±SD) | 72.9±9.5 | 69.9±10.5 | 0.121 |
| Female sex (n %) | 8(40.0) | 56(47.9) | 0.493 |
| Hypertension (n %) | 15(75.0) | 91(66.4) | <0.001 |
| Diabetes mellitus (n %) | 16(80.0) | 40(34.2) | 0.201 |
| Hyperlipidemia (n %) | 20(100%) | 20(17.1) | 0.044 |
| Atrial fibrillation (n %) | 16(80.0) | 26(22.2) | 1.0 |
| History of stroke( n %) | 19(95.0) | 1(0.85) | 0.273 |
| Tobacco use (current or past) (n %) | 12(60.0) | 22(18.8) | 0.045 |
| Occlusion artery |  |  | 0.472 |
| M1 (n %) | 6(30.0) | 51(43.6) |  |
| ICA terminus (n %) | 12(60.0) | 52(44.4) |  |
| Tandem (n %) | 2(10.0) | 14(12.0) |  |
| NIHSS (mean ±SD) | 18.9±2.7 | 16.4±6.2 | 0.072 |
| Baseline ASPECTS (mean ±SD) | 3.2±2.5 | 3.8±2.9 | 0.339 |
| iv tPA (n %) | 5(25.0) | 34(29.1) | <0.001 |
| mTICI score of 2b-3 (n %) | 17(85.0) | 12(10.3) | 0.463 |
| Time from onset to treatment, min(mean ±SD) | 104±92 | 102±46 | 0.894 |
| Time from groin puncture to reperfusion, min. (mean ±SD) | 60±57 | 55±25 | 0.697 |
| CBF<30% |  |  |  |
| Ischemic core, ml(mean ±SD) | 99.5±47.1 | 90.5±43.4 | 0.429 |
| Mismatch volume ml(mean ±SD) | 130.6±63.2 | 124.9±110.7 | 0.822 |
| Mismatch ratio(mean ±SD) | 2.6±0.9 | 2.5±1.7 | 0.817 |
| CBF<25% |  |  |  |
| Ischemic core, ml(mean ±SD) | 82.6±44.1 | 71.6±36.8 | 0.292 |
| Mismatch volume, ml(mean ±SD) | 140.4+113.1 | 139.7±63.9 | 0.991 |
| Mismatch ratio(mean ±SD) | 3.4±1.4 | 3.2±2.6 | 0.756 |
| CBF<20% |  |  |  |
| Ischemic core, ml(mean ±SD) | 65.2±39.0 | 54.7±35.1 | 0.263 |
| Mismatch volume, ml(mean ±SD) | 158.6±115.0 | 157.7±66.7 | 0.998 |
| Mismatch ratio(mean ±SD) | 4.8±4.3 | 4.6±2.8 | 0.87 |
